# Feasibility study of an alkaline-based chemical treatment for the purification of polyhydroxybutyrate produced by a mixed enriched culture

**DOI:** 10.1186/s13568-015-0096-5

**Published:** 2015-01-24

**Authors:** Yang Jiang, Gizela Mikova, Robbert Kleerebezem, Luuk AM van der Wielen, Maria C Cuellar

**Affiliations:** Department of Biotechnology, Delft University of Technology, Julianalaan 67, 2628 BC Delft, The Netherlands; Polymer Technology Group Eindhoven BV, De Lismortel 31, 5612 AR Eindhoven, The Netherlands

**Keywords:** Polyhydroxybutyrate, Alkaline treatment, Crystallinity, Thermal stability, Mixed cultures

## Abstract

This study focused on investigating the feasibility of purifying polyhydroxybutyrate (PHB) from mixed culture biomass by alkaline-based chemical treatment. The PHB-containing biomass was enriched on acetate under non-sterile conditions. Alkaline treatment (0.2 M NaOH) together with surfactant SDS (0.2 w/v% SDS) could reach 99% purity, with more than 90% recovery. The lost PHB could be mostly attributed to PHB hydrolysis during the alkaline treatment. PHB hydrolysis could be moderated by increasing the crystallinity of the PHB granules, for example, by biomass pretreatment (e.g. freezing or lyophilization) or by effective cell lysis (e.g. adjusting alkali concentration). The suitability of the purified PHB by alkaline treatment for polymer applications was evaluated by molecular weight and thermal stability. A solvent based purification method was also performed for comparison purposes. As result, PHB produced by mixed enriched cultures was found suitable for thermoplastic applications when purified by the solvent method. While the alkaline method resulted in purity, recovery yield and molecular weight comparable to values reported in literature for PHB produced by pure cultures, it was found unsuitable for thermoplastic applications. Given the potential low cost and favorable environmental impact of this method, it is expected that PHB purified by alkaline method may be suitable for other non-thermal polymer applications, and as a platform chemical.

## Introduction

Polyhydroxyalkanoates (PHAs) have received much attention as bio-based plastics that may contribute to future replacement of petroleum based plastics. Their performance ranges from stiff and brittle to soft and tough (Sudesh et al. 2000 and Laycock et al. 2013). The most common PHA is polyhydroxybutyrate (PHB), which has similar thermal and some mechanical properties (e.g. tensile strength) compared to isotactic polypropylene (Sudesh et al. 2000). In contrast to petroleum based plastics, PHA’s biodegradability in various natural environments makes them suitable as disposables for packaging, agricultural or medical applications (Williams and Martin 2002, Bucci et al. 2005, Markets and Markets, 2013). The fact that more and more varieties of PHAs have been discovered and/or synthesized suggests that PHAs are not limited to thermoplastic applications. Moreover, PHA derivatives such as hydroxy fatty acid monomers may serve as chiral building blocks for the production of biochemicals and the methyl esters of their monomers could be used as a biofuel (Chen, 2009).

Chen (2009) summarized the current status of commercial PHA production. Many types of commercial PHAs are available on the market. For example, Polyhydroxybutyrate-co-hydroxyvalerate (PHBV) can be synthesized by pure culture of either *Ralstonia eutropha* or recombinant *E. coli* from glucose and propionic acid. Middle chain length PHAs, such as polyhydroxyhydroxyhexanoate (PHHx), can be produced by pure culture of *Pseudomonas putida*. Despite of the above mentioned advantages of PHAs compared to conventional petroleum based plastics their large scale application is still constrained by their high price in the market. Economic evaluations of the PHA production process identified the following cost drivers (Choi and Lee, 1997; van Wegen et al. 1998): (a) raw materials (fermentation feedstock), (b) downstream processes for product recovery and purification, and (c) costs associated to maintaining a pure culture during the fermentation (e.g. fermentor costs and energy required for sterilization). Several studies have integrated the PHA production process with wastewater treatment with a dynamic feast-famine enrichment system, aiming at intracellular PHB content up to 90% (Johnson et al. 2009), in order to reduce the cost from raw material and energy consumption aspects (reviewed by Dias et al. 2006). Recent results showed that such process is capable of producing PHAs as good as the current pure-culture process in terms of intracellular PHAs content and biomass specific PHAs production rates (Jiang et al. 2012). However, the challenge in terms of cost reduction in downstream process still remains.

PHAs are present in microorganisms as hydrophobic and water insoluble inclusion bodies which need to be separated from cell material. Plenty of techniques for PHA recovery and purification from pure cultures have been evaluated in literature and reviewed by Jacquel et al. (2008) and by Kunasundari and Sudesh (2011). The conventional organic solvent based purification method is still the best in terms of final product purity and recovery yield, although organic solvents may generate environmental issues (Ramsay et al. 1994; de Koning and Witholt, 1997). Several less toxic organic solvents have been reported for PHAs extraction (summarized in Jacquel et al. 2008; Kunasundari and Sudesh 2011; Riedel et al. 2013). Most of those solvents are specific for middle chain length PHAs purification, instead of short chain length PHAs (e.g. PHB, PHV) (Jiang et al. 2006; Elbahloul and Steinbüchel, 2009; Terada and Marchessault 1999). Nevertheless, short chain length PHAs are usually the main products when wastewater is used as feedstock (Dionisi et al. 2005; Bengtsson et al. 2008; Albuquerque et al. 2010; Jiang et al. 2012). Moreover, solvents such as 1, 2-proplene bicarbonate, require high temperature (>140°C) during the purification process, which typically leads to high energy consumption (Fiorese et al. 2009; Riedel et al. 2013).

Removal of cell materials by alkaline treatment was considered more economically feasible by Choi and Lee (1997, 1999) as compared to an organic solvent based PHA purification process. The alkaline treatment method has been widely reported in literature for pure cultures, resulting in purity and recovery yield as high as 98% and 97%, respectively (Choi and Lee 1999, Mohammadi et al. 2012a, b). An open culture process is based on the enrichment of a mixture of different microorganisms; it is unclear whether alkaline treatment can equally remove cell materials from microorganisms from enriched mixed cultures. Furthermore, the chemicals used in the treatment could degrade the PHA granules, as well as negatively influence the thermal stability of PHAs during processing as thermoplastics (Kim et al. 2006).

The fate of PHAs during alkaline treatment and the thermal stability of the chemically treated PHA have hardly been reported. Moreover, only few studies have been published on recovery and purification of PHAs from mixed cultures (Serafim et al. 2008). In this study, the feasibility of the alkaline method for recovery and purification of PHB obtained from mixed cultures was evaluated. This study focused on the PHA degradation during the chemical treatment and on product properties such as molecular weight and thermal stability. PHB recovery and purification by extraction with dichloromethane was used for comparison purposes.

## Material and methods

### Biomass preparation and PHB recovery

The biomass used in this study was obtained from a 2 L sequencing batch reactor (SBR) fed with acetate under feast-famine condition. The composition of the working medium was: 125 mM NaAc · 3H_2_O, 3.93 mM NH_4_Cl, 1.87 mM KH_2_PO_4_, 0.42 mM MgSO_4_ · 7H_2_O, 0.54 mM KCl, 1.13 ml/L trace elements solution according to Vishniac and Santer (1957) and 3.71 mg/L allythiourea (to prevent nitrification). The operational conditions of the bioreactor were 30°C, pH 7, 1 day sludge retention time (SRT) and hydraulic retention time (HRT) and 18 h cycle length. The length of the feast phase was about 2.5 h during the steady state. The PHB was the sole storage polymer produced due to the fact that acetate was the sole carbon source. The biomass was collected at the end of the feast phase, when the cellular PHB content was between 62 wt% and 72 wt%. The dominant bacterial species in the SBR operated under such condition was *P. acidivorans*, a gram-negative bacterium (Jiang et al. 2011).

Fresh biomass from bioreactor was collected by centrifugation (Heraeus, Germany) at 10000 g for 10 min at room temperature. The supernatant was removed and the pellet was resuspended with Milli-Q water to reach a final biomass concentration of approximately 20 g/L. 10 mL of this biomass suspension was used for PHB recovery. Two types of chemicals were applied either solely or together to remove the cell materials: (1) alkalis (NaOH at concentrations varying between 0.02 M and 1 M, or 0.2 M NH_4_OH), and (2) surfactant (SDS at concentrations varying between 0.025% and 0.2%). The biomass suspension with the added chemicals was incubated in 50 mL tubes at 200 rpm and 30°C for 1 hour unless otherwise stated. The suspension was subsequently centrifuged at 10000 g for 10 min at 4°C. The supernatant was separated from the pellet and collected for soluble polymer or monomer measurements. The pellet was washed twice with Milli-Q water and dried at 60°C overnight.

Besides fresh biomass, pre-treated biomass was also evaluated in this study. The fresh biomass pellet collected after centrifugation was subjected to either freezing at −20°C or lyophilization. The same chemical treatment procedures as for the fresh biomass were applied to the pre-treated biomass in order to study the influence of pre-treatment on the PHB recovery. Lyophilized biomass was additionally used for solvent extraction for comparison purposes. The PHB was firstly purified by dichloromethane, following the procedure described in Ramsay et al. (1994). Further purification was achieved by dissolving 1 wt% of PHB in chloroform at 60°C for 50 min. The chloroform sample was subsequently slowly poured into cold ethanol (10 times volume to chloroform) while stirring rigorously. The precipitate was filtered of the solution, washed with ethanol and vacuum dried at 50°C.

The setup of all the experiments in this study is summarized in the Table 1. All experiments were performed in at least duplicate.

**Table 1:** **Summary of all experiments conducted in this study**

**Chemical**	**Concentration**	**Biomass state**	**Time**	**Initial PHB content**	**Purity**	**Recovery**	**Mass balance**	**HB/PHB** ^**c**^
[−]	[M;w/v%]	[−]	[h]	[wt%]	[%]	[%]	[%]	[%]
NaOH	0.02	Fresh	1	71.8 ± 5.7	77.3 ± 4.0	92.2 ± 4.2	−1.0 ± 3.7	97.9 ± 5.3
NaOH	0.05	Fresh	1	72.7 ± 7.1	84.0 ± 1.4	94.7 ± 3.2	−3.9 ± 2.9	96.7 ± 4.2
NaOH	0.10	Fresh	1	65.3 ± 3.1	83.8 ± 4.4	98.0 ± 1.4	−1.0 ± 1.4	92.1 ± 8.5
NaOH	0.20	Fresh	1	69.4 ± 1.1	86.6 ± 3.0	96.7 ± 1.9	−0.7 ± 2.6	97.5 ± 16.7
NaOH	0.20	Fresh	0.3	68.6 ± 0.7	87.3 ± 2.2	96.4 ± 2.6	−2.9 ± 2.4	85.9 ± 14.8
NaOH	0.20	Fresh	0.5	68.6 ± 0.7	88.8 ± 0.8	98.5 ± 1.8	−0.7 ± 1.8	90.9 ± 18.9
NaOH	0.20	Fresh	3	68.6 ± 0.7	92.1 ± 0.8	93.5 ± 2.4	−1.5 ± 0.6	92.0 ± 3.2
NaOH	0.40	Fresh	1	65.3 ± 3.1	87.9 ± 5.4	95.2 ± 3.7	0.7 ± 3.1	89.9 ± 4.8
NaOH	0.70	Fresh	1	65.3 ± 3.1	89.7 ± 5.8	90.9 ± 5.0	0.4 ± 4.4	89.4 ± 7.1
NaOH	1.00	Fresh	1	65.3 ± 3.1	90.6 ± 4.7	85.6 ± 2.3	−0.3 ± 2.1	89.1 ± 4.0
NH_4_OH	0.20	Fresh	1	68.6 ± 0.7	62.6 ± 2.8	63.3 ± 16.4	−3.9 ± 0.9	36.3 ± 10.9
SDS	0.20	Fresh	1	68.0 ± 0.0	79.0 ± 1.4	63.5 ± 0.7	3.6 ± 0.6	14.0 ± 1.4
NaOH + SDS	0.20 + 0.025	Fresh	1	66.1 ± 2.2	94.9 ± 2.6	92.6 ± 6.9	−2.7 ± 3.8	94.2 ± 6.4
NaOH + SDS	0.20 + 0.050	Fresh	1	66.1 ± 2.2	96.9 ± 1.3	93.5 ± 4.8	−2.4 ± 2.4	92.4 ± 3.1
NaOH + SDS	0.20 + 0.100	Fresh	1	66.1 ± 2.2	98.3 ± 0.5	91.5 ± 5.9	−3.9 ± 4.8	96.3 ± 4.8
NaOH + SDS	0.20 + 0.200	Fresh	1	66.1 ± 2.2	99.1 ± 0.5	91.0 ± 4.9	−3.1 ± 1.9	92.5 ± 5.0
NaOH	0.20	Freezing	1	65.9 ± 2.4	94.1 ± 3.5	95.6 ± 2.5	−2.9 ± 2.1	94.3 ± 5.4
NaOH	0.20	Freeze dried	1	69.9 ± 2.2	95.9 ± 3.7	95.5 ± 0.6	−3.2 ± 0.8	98.8 ± 0.9
NH_4_OH	0.20	Freeze dried	1	69.9 ± 2.2	87.4 ± 2.1	95.0 ± 1.8	−3.9 ± 0.9	87.1 ± 12.9
SDS	0.20	Freeze dried	1	69.9 ± 2.2	93.5 ± 4.1	93.7 ± 2.2	−3.1 ± 1.6	91.3 ± 8.7
CH_2_Cl_2_	30^a^	Freeze dried	o/n^b^	72.2 ± 0.4	97.6	55.9	ND	ND

^a^30 times of TSS.

^b^Overnight.

^c^Fraction of hydrolyzed monomer in total polymer in the supernatant.

### Analytical methods

In order to evaluate the PHB mass balance of all experiments, the PHB quantity in fresh biomass, in final products and in the supernatant were determined. The PHB content in the biomass and in the final products was determined by gas chromatography (GC) according to the method described in Johnson et al. (2009). Commercial PHB (SigmaAldrich, the Netherlands) was used as standard. Based on the PHB mass present in the biomass (*PHA*_*initial*_) and the dried pellet (*PHA*_*end*_), the recovery yield was calculated by equation 1:

1 $$ Recovery=\frac{PH{A}_{end}}{PH{A}_{initial}}\cdot 100\%\kern2em \left[\mathrm{g}/\mathrm{g}\right] $$

The PHB losses in the supernatant after chemical treatment (*PHB*_*supernatant*_) was analyzed by gas chromatography (GC) with a modified procedure: 0.5 ml of the supernatant from chemical treatment was used for PHB concentration analysis. Commercial PHB mixed with 0.5 ml of chemical solution for PHB purification was used as standard. The remaining procedures were the same as described in Johnson et al. (2009). The potential by-products of chemical treatment (e.g. hydrobutyric acid, HB and crotonic acid, CA) (Yu et al. 2005) were analyzed by high-performance liquid chromatography (HPLC) with a BioRad Animex HPX-87H column and a UV detector (Waters 484, 210 nm). The mobile phase, 1.5 mM H_3_PO_4_ in Milli-Q water, had a flow rate of 0.6 mL/min and a temperature of 59°C.

The overall mass balance was calculated by equation 2:

2 $$ MassBalance=\frac{\left(PH{A}_{end}+PH{B}_{supernatant}+C{A}_{supernatant}-PH{A}_{initial}\right)}{PH{A}_{initial}}\times 100\%\kern2em \left[\mathrm{g}/\mathrm{g}\right] $$

where, *PHB*_*supernatant*_ means the total PHB loss within the supernatant measured by GC and *CA*_*supernatant*_ indicates the identified crotonic acid in the supernatant by HPLC. As a consequence, a closer value to 0% indicates a better mass balance. In this study, most of the experiments had mass balance errors smaller than 5% (see Table 1).

A degree of PHA degradation was defined as the fraction of HB or CA concentration over total initial PHB mass in the biomass (equation 3 or 4).

3 $$ HB/PH{B}_{initial}=\frac{H{B}_{supernatant}}{PH{A}_{initial}}\cdot 100\%\kern2em \left[\mathrm{g}/\mathrm{g}\right] $$

or,

4 $$ CA/PH{B}_{initial}=\frac{C{A}_{supernatant}}{PH{A}_{initial}}\cdot 100\%\kern2em \left[\mathrm{g}/\mathrm{g}\right] $$

Chemical PHB degradation may occur either randomly in the middle of the polymer chain or from the end of the polymer chain. The GC method measured the overall lost PHB in the supernatant in terms of both soluble PHB oligomers and HB monomer, while HPLC method only quantified the HB monomers. A ratio between soluble HB monomer and overall PHB in the supernatant was used to assess the chemical PHB degradation mechanism (equation 5). A higher value (close to 1) indicates that HB is sole product of PHB degradation, suggesting PHB is degraded from the end of the polymer chain. Otherwise, PHB is more likely hydrolyzed by chemicals randomly from the middle of the chain, generating oligomers as products.

5 $$ HB/PH{B}_{supernatant}=\frac{H{B}_{supernatant}}{PH{A}_{supernatant}}\cdot 100\%\kern2em \left[\mathrm{g}/\mathrm{g}\right] $$

### Fourier transform infrared spectroscopy (FTIR)

The composition and the crystallinity of dry pellets were examined using a spectrum 100 FT-IR spectrometer (PerkinElmer). The solid powders were pressed on a germanium crystal window of a microhorizontal ATR for measurement of single reflection and absorption of infrared by the specimens.

### Thermal stability

Around 100 mg of an untreated biomass, PHB isolated from biomass by a chemical or an organic solvent treatment and/or a commercial PHB (Tianan, China) were isothermally treated in a compression molding machine (Dr Collins) at 170°C for a certain period of time (1, 3, 5, 10 and 15 min). The molecular weight of PHB before and after the thermal treatment was determined by a size exclusion chromatography (SEC). For SEC analysis, around 3 mg of a sample was dissolved in 1 ml hexafluoroisopropanol (HFIP) at room temperature overnight. The sample was subsequently filtered using 0.2 μm filter. Molar mass distribution was determined using a Waters model 510 pump and a Waters 712 WISP chromatograph with PL-gel mix D columns (300 × 7.5 mm, Polymer Laboratories). HFIP was used as an eluent with a flow rate of 1 ml/min. The system was calibrated with PMMA standards.

The thermal degradation rate can be expressed by the equation 6 (Grassie et al. 1984a, b):

6 $$ \left(\frac{1}{Pn,t} - \frac{1}{Pn,0}\right) = {k}_D\ t\kern2em \left[1/\mathrm{s}\right] $$

where, P_n_,t and P_n,0_ are number average degrees of polymerization at time t and time 0 s, respectively. The rate constant (k_D_) was determined from the slope of the equation 6 function. P_n_,t and P_n,0_ were calculated using number average of molecular weight (M_n_) in time t and time 0 s according to equations 7a and 7b.

7a $$ {P}_{n,t} = \frac{M_{n,t}}{M_m}\kern3em \left[\left(\mathrm{g}/\mathrm{mol}\right)/\left(\mathrm{g}/\mathrm{mol}\right)\right] $$

7b $$ {P}_{n,0} = \frac{M_{n,0}}{M_m}\kern3em \left[\left(\mathrm{g}/\mathrm{mol}\right)/\left(\mathrm{g}/\mathrm{mol}\right)\right] $$

*M*_*m*_ is the molecular weight of a PHB monomer unit, i.e. 86.09 g/mol.

## Results

### PHB recovery and purification

Alkalis and surfactant were two chemicals used in this study in order to purify and recover PHB from fresh biomass. Initially sole NaOH treatments with different concentration and treatment time were conducted (see Table 1). The final product purity increased by increasing NaOH concentration or by the prolonged treatment time, but the recovery yield was negatively influenced by those two parameters. On the basis of the final product purity and recovery yield, the treatment with 0.2 M NaOH for 1 h was chosen as the standard condition (see Table 1). Under this standard condition, the final product purity and the recovery yield can reach 87% and 97%, respectively. In order to improve the purity from the standard condition, and to favor the sustainability of the process, different chemicals combinations were tested. With the purpose of improving the purity, surfactant was added to the standard condition to remove the cell materials further. With additional dosage of SDS to our standard condition, the purity can be improved up to 99% with a slight decrease in recovery yield (91%). NH_4_OH was aimed at replacing NaOH, because it is potentially easier to be recycled than NaOH (van Hee et al. 2005). However, significant decrease was observed in both purity (to 63%) and recovery yield (63%) when treating fresh biomass with 0.2 M NH_4_OH.

Besides recovering PHB from fresh biomass, the effect of pre-treatment such as lyophilization or freezing, was also studied. These pre-treatments led to a higher purity in all cases and an improved recovery yield in sole SDS and NH_4_OH treatment (see Table 1). For comparison purposes, recovery and purification by solvent extraction was also conducted in this study. Extraction with dichloromethane reached 98% purity from lyophilized biomass. However, the recovery yield was very low (55%) in this study.

### Thermal stability of purified PHB

In order to utilize PHAs as thermoplastics, thermal stability is a crucial parameter. Thermoplastic polymers are usually processed at temperatures at least 10°C above their melting point and typical residential time in an extruder does not exceed one minute. The processing temperature of PHB is usually between 170 and 180°C. Therefore, the thermal stability of the samples was studied in terms of PHB degradation during the first minute at 170°C.

**Figure 1:**
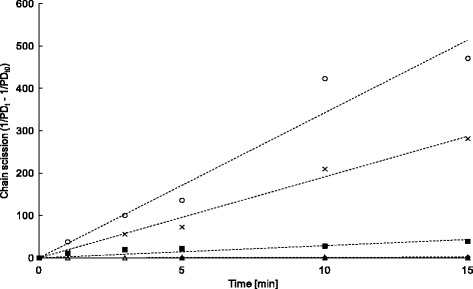
**The effect of chemical treatment on thermal stability of commercial PHB (Tianan) and PHB isolated from biomass represented here by polymer chain scission (** ***1/P***_***n,t***_ ***– 1/P***_***n,0***_ **) as a function of time at 170°C.** Water content in the samples was in between 0.01 and 0.02 wt%. The numbers in brackets represent PHB purity. PHB purified by 0.2 M NaOH (empty circle, 85% pure); PHB purified by 0.2 M NaOH and 0.2% SDS (cross, 95% pure); Unpurified PHB within biomass (solid square, 67% pure); PHB purified by solvent (empty triangle, 99% pure); Commercial PHB (solid diamond, 99% pure).

Number average of molecular weight (*M*_*n*_) of PHB as a function of time during the thermal treatment is shown in Table 2. PHB isolated from biomass by a solvent method and the commercial PHB showed the highest thermal stability with less than 7% *M*_*n*_ drop within the first minute of the treatment (*ΔM*_*n,1*_). The resulting molecular weight after the processing was still acceptable for a plastic application (*M*_*n*_ > 169 kg/mol). The sample purified by 0.2 M NaOH or by 0.2 M NaOH and 0.2% SDS showed much more pronounced molecular weight decrease (*ΔM*_*n,1*_ > 70%). The consequent molecular weights were below 45 kg/mol. As compared to the chemically purified PHB, the degradation of the polymer in the untreated biomass was less detrimental (*ΔM*_*n,1*_ = 62%). The rate of the polymer chain scission, i.e. the degradation rate constant (*k*_*D*_), was calculated from the slope of the kinetic function shown in Figure 1. Thermal stability results at 170°C are summarized in Table 3, in terms of a ratio between *k*_*D*_ of a specific sample and *k*_*D*_ of the commercial PHB reference (*k*_*D,ref*_). It can be observed that both, the untreated biomass and the chemically purified PHB showed significant deterioration in terms of a faster degradation rate. On the other side, the solvent isolated PHB performed even better than the commercial sample.

**Table 2:** **Molecular weight (number average** ***M***_***n***_ **and weight average** ***M***_***w***_ **) and molecular weight change** $\left(\left(\frac{{\boldsymbol{M}}_{\boldsymbol{n},\boldsymbol{0}}\hbox{-} {\boldsymbol{M}}_{\boldsymbol{n},\boldsymbol{t}}}{{\boldsymbol{M}}_{\boldsymbol{n},\boldsymbol{0}}}\right)\mathbf{x}\ \mathbf{100}\right)$ **of various PHB samples as a function of thermal treatment at 170°C.**

**Sample**	**Chemical treatment**	**PHB purity [wt.%]**	**Time of thermal treatment at 170°C [min]**	***M***_***n***_ **[kg/mol]**	***M***_***w***_ **[kg/mol]**	$\left(\frac{{\boldsymbol{M}}_{\boldsymbol{n},\boldsymbol{0}}\hbox{-} {\boldsymbol{M}}_{\boldsymbol{n},\boldsymbol{t}}}{{\boldsymbol{M}}_{\boldsymbol{n},\boldsymbol{0}}}\right)\mathbf{x}\ \mathbf{100}$ **[%]**
Commercial PHB	-	99	0	182	647	0
			1	169	583	7
			3	175	541	4
			5	119	391	35
			10	150	435	18
			15	135	373	26
PHB from biomass	-	67	0	135	224	0
			1	51	111	62
			3	33	62	76
			5	30	42	78
			10	25	34	81
			15	19	25	86
	Solvent	99	0	915	1755	0
			1	883	1731	3
			3	824	1573	10
			5	771	1562	15
			10	516	1144	44
			15	560	1255	39
	0.2 M NaOH	85	0	119	315	0
			1	19	39	84
			3	8	13	93
			5	6	8	95
			10	2	3	98
			15	1.8	2.2	98
	0.2 M NaOH + 0.2% SDS	95	0	163	484	0
			1	45	73	72
			3	14	23	91
			5	11	20	93
			10	4	8	98
			15	3	4	98

Water content in the samples was in between 0.01 and 0.02 wt.%.

**Table 3:** **Thermal degradation rate constants (** ***k***_***D***_ **) of various PHB samples at 170°C and thermal degradation rate constants relative to the commercial PHB reference (** ***k***_***D,ref***_ **) as a function of chemical treatment, purification method and purity**

**Sample**	**Chemical treatment**	**PHB purity [wt.%]**	**k**_**D**_ **10** ^**−6**^ **[1/s]**	**k**_**D**_ **/k**_**D,ref**_ **10** ^**−6**^ **[1/s]**
Commercial PHB	-	99	0.18 ± 0.02*	1.0
	0.2 M NaOH	99	1.40 ± 0.10	8.0
	0.2% SDS	99	0.80 ± 0.10	4.0
PHB from biomass	-	67	5.40 ± 0.80	30.0
	Solvent	99	0.08 ± 0.01	0.4
	0.2 M NaOH	85	54.00 ± 5.00	300.0
	0.2 M NaOH + 0.2% SDS	95	29.00 ± 2.00	161.0

*k_*D*_ of dried commercial PHB was used as a reference (*k*_*D,ref*_).

Water content in all samples was in between 0.01 – 0.02 wt%.

### PHB degradation by alkalis

**Figure 2:**
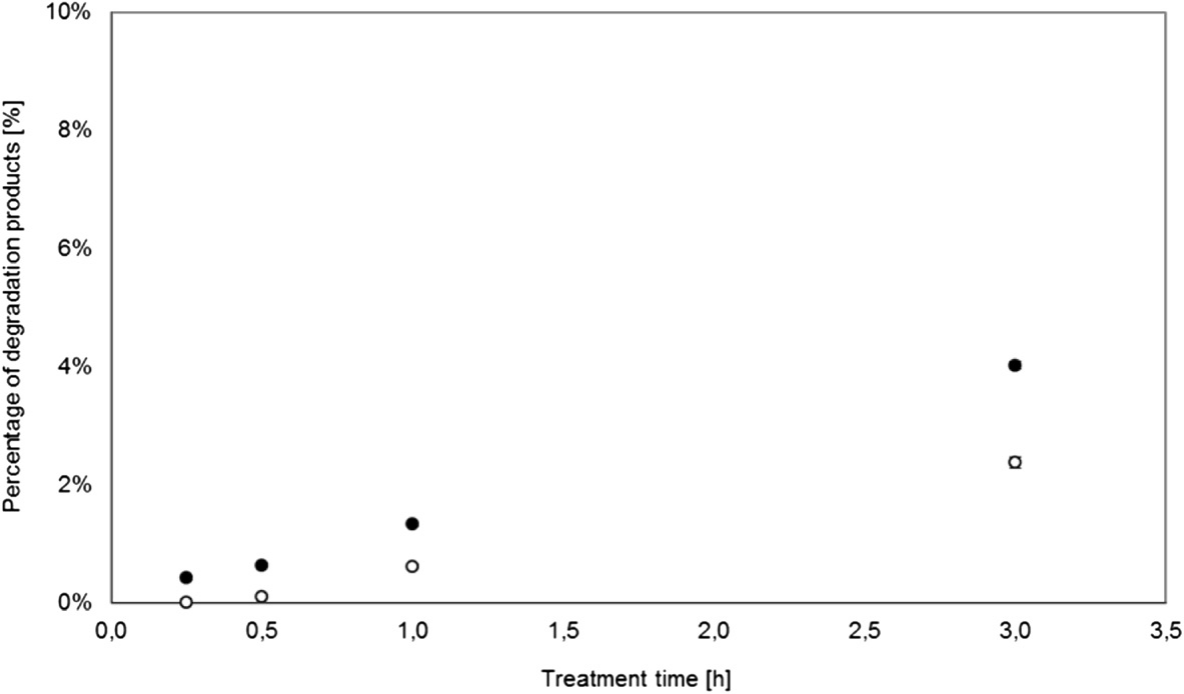
**The relation between monomers production from PHB and NaOH treatment time.** The fraction of two monomer products, hydroxybutyric acid (HB) and crotonic acid (CA) over total initial PHB (equations 3 and 4) are indicated by solid circle and empty diamond, respectively. The experiment was performed with fresh biomass at 0.2 M NaOH and 30°C in duplicate. Initial PHB content was 68%.

**Figure 3:**
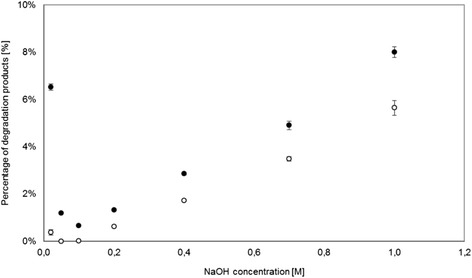
**Fraction of degradation products, HB (solid round) and CA (empty diamond), over total initial PHB (equations** **3** **and** **4** **).** The experiment was performed with fresh biomass at 30°C for 1 hour in duplicate. Initial PHB content was 68%.

The thermal instability of PHB purified by alkalis based method could be due to PHB hydrolysis. As it has been reported in Yu et al. (2005), abiotic hydrolysis of PHB by alkalis was observed in this study as well. Both HB monomer and CA were found as PHB hydrolysis products. Our data showed that the PHB degradation by NaOH in the fresh biomass was dependent on the treatment time and NaOH concentration. The hydrolysis products concentration showed linear relation with NaOH treatment time (Figure 2), while the relation between the NaOH concentration and the hydrolyzed products concentration is non-linear (Figure 3). In the tested NaOH concentration range, the HB monomer decreased with the increasing NaOH concentration before 0.1 M NaOH and then increased with NaOH concentration. For an initial PHB content of 68%, at the standard condition in this study (i.e. 0.2 M NaOH treatment for 1 h with fresh biomass), about 1.3% of initial PHB was hydrolyzed into HB monomer and about 0.6% of initial PHB was converted to CA.

**Figure 4:**
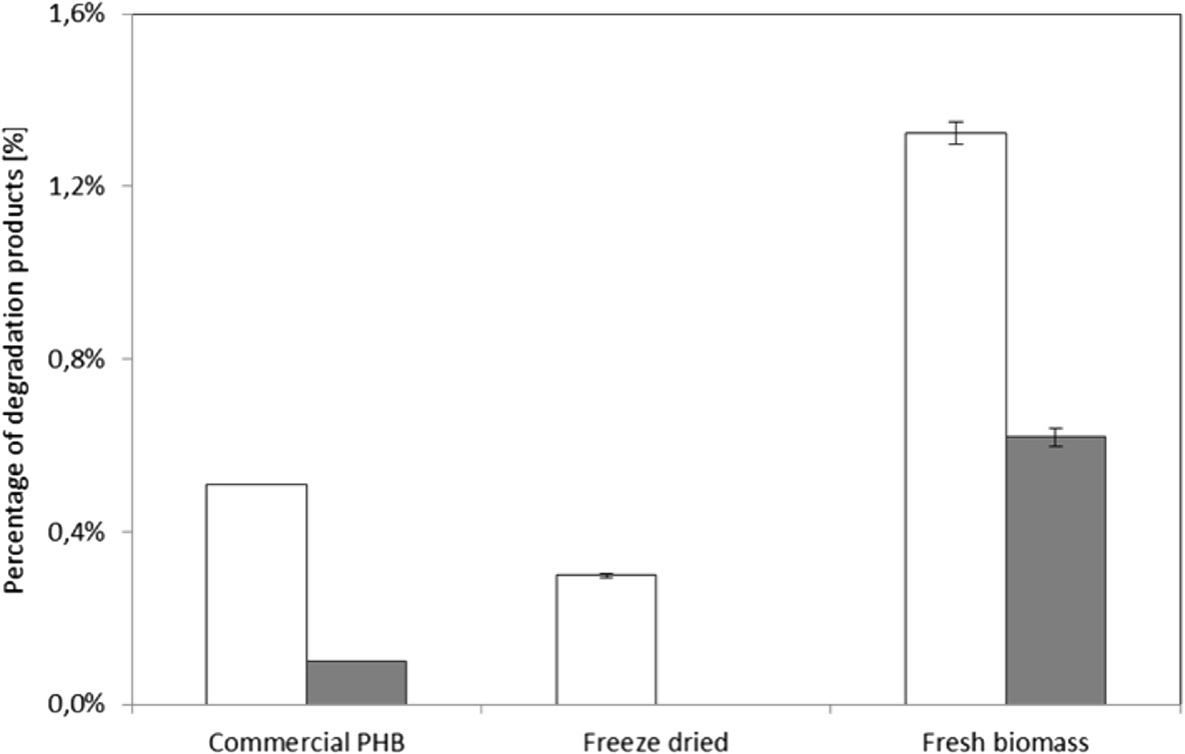
**The influence of lyophilization on the PHB degradation by NaOH, expressed as fraction of degradation products over total initial PHB (equations** **3** **and** **4** **).** White color indicates HB and gray color represents CA. Samples were treated with 0.2 M NaOH for 1 hour at 30°C in duplicate. Initial PHB content was 68%.

The pre-treatment step also showed some influence on the PHB hydrolysis. Much less HB or CA was produced after lyophilization or freezing pre-treatment (Figure 4).

The spectrum of hydrolysis products in the supernatant can be used as an indication of the chemical PHB degradation mechanism (equation 5). When the biomass with or without pre-treatment was treated by NaOH, *HB/PHB*_*supernatant*_ ratio was always close to 100% (see Table 1). The closed mass balance in this study suggested that no other forms of soluble PHB oligomers were formed during NaOH treatment.

### FTIR spectra

The effect of NaOH concentration and pre-treatment on PHB hydrolysis was investigated further by evaluating the crystallinity state of several samples through FTIR analysis (Xu et al. 2002; Yu and Chen 2006). An intensity ratio of the absorbance at 1230 cm^−1^ to that at 1453 cm^−1^ was used to calculate the polymer crystallinity index (CI, Xu et al. 2002). Larger CI value corresponds to higher crystallinity whilst smaller values reflect lower crystalline portion. As can be seen from Table 4, both chemical treatment and pre-treatment process show influence on PHB CI value. NH_4_OH treated sample showed the lowest crystallinity compared to the rest of the samples.

**Table 4:** **Crystallinity index (CI = A**_**1230**_ **/A**_**1453**_ **)**

**Biomass status**	**Chemicals**	**CI**
Commercial PHB	-	4.7
Lyophilized	CH_2_Cl_2_	5.7
Lyophilized	SDS	4.7
Lyophilized	NaOH	4.5
Fresh biomass	NaOH + SDS	4.4
Lyophilized	NH_4_OH	4.2
Fresh biomass	NaOH	3.8
Lyophilized	-	2.9
Fresh biomass	NH_4_OH	2.1

Larger value means that PHB is at a more crystallinity status and smaller value means that PHB is at a more amorphous status.

**Figure 5:**
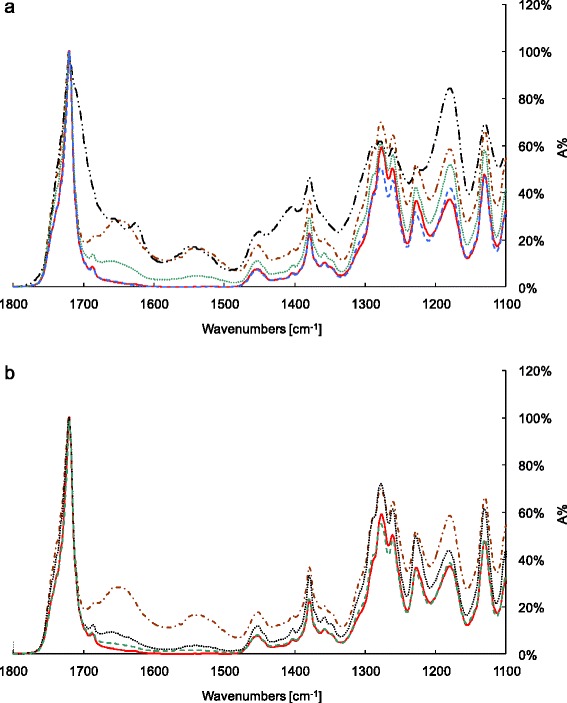
**IR spectra of PHB-containing biomass treated with different chemicals. (a)** Chemical treatment with fresh biomass. Freeze dried biomass, brown dash dot line; 0.2 M NH_4_OH treatment, black dash double dots line; 0.2 M NaOH treatment, green dot line; 0.2 M NaOH and 0.25 w/v% SDS treatment, blue dash line; Commercial PHB, red line. **(b)** Chemical treatment with freeze dried biomass. Freeze dried biomass, brown dash dot line; 0.2 M NH_4_OH treatment, black dot line; 0.2 M NaOH treatment, green dash line; Commercial PHB, red line. All of the treatments were conducted at 30°C for 1 hour. The absorbance intensity was normalized by the intensity of absorbance at 1720 cm^−1^.

FTIR can also be used to qualitatively detect both PHB and proteins in the final products (Yu and Chen 2006). Therefore, all purified products were analyzed by FTIR, together with commercial PHB as control of PHB absorbance, and lyophilized biomass as a control of both PHB and protein absorbance. Figure 5a shows the spectrum of PHB from fresh biomass purified by different chemicals in comparison with commercial PHB and lyophilized biomass. The absorption at 1720 cm^−1^ and 1278 cm^−1^ respectively indicates C = O stretch and C-O stretch of the ester bonds. They both represent the presence of PHB. The absorption peaks at 1650 cm^−1^ and 1540 cm^−1^ represent amide I and amide II band in proteins. As can be seen in Figure 5, the commercial PHB and the PHB purified by NaOH-SDS mixture show highly similar spectra. In contrast, proteins were detected in all other samples.

## Discussion

### PHB recovery and purification

In this study, a high PHB purity was obtained from fresh biomass by treatment with alkali and surfactant. In principle, both alkali and surfactant can react with lipid and proteins, solubilizing the cell wall material and releasing the intracellular contents. Our results suggest that sole NaOH treatment can lyse cells but it is insufficient to remove all cell materials. Under our standard condition (0.2 M NaOH, for 1 hour at 30°C), still about 13.4% of cell material impurities remained in the final pellets. Those remaining impurities are likely water insoluble proteins and lipids. We observed that those hydrophobic impurities can be effectively removed by combined NaOH and SDS treatment. Higher SDS concentrations resulted in a higher final purity, likely due to micelle formation by SDS. Once the SDS concentration approached its critical micelle concentration (CMC), which is between 0.17-0.23 w/v%, more proteins and lipids were removed. However, SDS micelles might also solubilize PHB granules. Indeed, our data showed that PHB recovery yield decreased at high SDS concentration (Table 1).

The hydroxide ion concentration was also observed to have influence on cell materials removal and PHB recovery. In the case of NH_4_OH and low NaOH concentration, for example, both purity and recovery were observed to be lower than at the standard condition. Since NH_4_OH is a weak base, at the same solution concentration the amount of dissociated hydroxide ion from NH_4_OH is much lower than for NaOH (about 100 times less). In fact, samples treated by 0.2 M NH_4_OH and 0.02 M NaOH displayed the lowest purity in this study (respectively 63% and 77%, see Table 1). Next to a decreased removal of cell materials, treatment at 0.2 M NH_4_OH showed more severe PHB degradation, which resulted in a lower recovery yield. This may be related to the PHB granules crystallinity status, which is discussed in the next section.

PHA recovery by chemical treatment has been widely reported in literature, but to our knowledge, on pure cultures only. The results are very diverse (Table 5). Considering the variability across studies in terms of microorganism, cell pre-treatment, temperature, initial PHB content and chemical concentration, among others, and their lack of PHB mass balance data, it is difficult to compare these results directly to our observations. Here we focus on the studies performed on fresh biomass, because at production scale it might be preferred to avoid any pre-treatment step.

**Table 5:** **Literature comparison**

**Bacteria species**	**Biomass status**	**Chemical**	**Concentration**	**Initial PHA content**	**Purity**	**Recovery**	**Reference**
*E. coli(rec)*	Frozen	NaOH	0.1	77%	91%	90%	Choi and Lee (1999)
*E. coli*	Frozen	SDS	0.5%	77%	98%	87%	Choi and Lee (1999)
*E. coli*	Frozen	NH_4_OH	0.1	77%	85%	95%	Choi and Lee (1999)
*E. coli*	Frozen	H_2_SO_4_	0.1	77%	79%	87%	Choi and Lee (1999)
*C.necator*	Lyophilized	NaOH	0.1	38%	97%	97%	(Mohammadi et al. 2012a, b)
*C.necator*	Lyophilized	NaOH	0.1	60%	80%	90%	Anis et al. (2012)
*C.necator*	Lyophilized	NH_4_OH	0.1	60%	60%	62%	Anis et al. (2012)
*Comamonas*	Lyophilized	NaOH	0.05	34%	89%	97%	(Mohammadi et al. 2012a, b)
*R.eutropha*	Lyophilized	NaOH	N.D.	70%	78%	45%	Yang et al. (2011)
*R.eutropha*	Lyophilized	SDS	5%	70%	90%	81%	Yang et al. (2011)
*R.eutropha*	Lyophilized	SDS	1%	50%	87%	N.D.	Ramsay et al. (1990)
*P.putida*	Lyophilized	NaOH	0.1	20%	40%	95%	Jiang et al. (2006)
*E. coli*	Oven dried	NaOH + SDS	0.1 + 10%	60%	87%	96%	Peng et al. (2013)
*R.eutropha*	Fresh	SDS	0.5%-20%	75%	97%	92%	Kim et al. (2003)
*R.eutropha*	Fresh	H_2_SO_4_	1	60%	76%	94%	Yu and Chen (2006)
*A.vinelandii*	Fresh	NH_3_	1	84%	94%	N.D.	Page and Cornish (1993)
*C.nector*	Fresh	NaOH	0.1	68%	84%	91%	Anis et al. (2013)
*E. coli*	Fresh	NaOH	0.2	79%	97%	91%	Choi and Lee (1999)
P.acidivorans*	Fresh	NaOH	0.2	68%	89%	97%	This study
P.acidivorans*	Fresh	NH_4_OH	0.2	68%	65%	78%	This study
P.acidivorans*	Lyophilized	NH_4_OH	0.2	68%	87%	96%	This study
P.acidivorans*	Fresh	NaOH + SDS	0.2 + 0.2%	68%	99%	95%	This study

*Dominant bacterial species in the mixed culture at the cultivation conditions of this study.

Choi and Lee (1999) reported that direct treatment of fresh recombinant *E.coli* by 0.2 M NaOH can result in 97% purity and 91% recovery. This is the best result described for sole NaOH treatment method. The major difference between their research and our study is that pure culture of recombinant bacteria were used in their research in contrast to mixed culture in our study. It is possible that some microorganism species in the mixed culture biomass are not efficiently treated by NaOH. Anis et al. (2013), for example, treated wet biomass of recombinant *C. necator* by 0.1 M NaOH, resulting in final purity (84%) and recovery yield (91%) more similar to our observations.

Regarding studies with sole surfactant treatment, Kim et al. (2003) applied SDS to *Ralstonia eutropha* cells, but additional heating at 121°C and washing steps were required to remove proteins and achieve a final purity of 97%. Interestingly, their PHB recovery (>92%) was remarkably higher than our results (63%, see Table 1). This suggests that temperature plays a significant role in the interaction between SDS and PHB − for example, due to altered critical micelle concentration (Bayrak 2003) − resulting in less PHB loss with the supernatant.

The synergistic effect of alkalis and surfactants on PHB recovery and purification has not been well studied yet. Peng et al. (2013) combined SDS and NaOH for PHB purification of dried cells, resulting in lower purity (87%) but comparable recovery yield (96%) as in our study (99% and 95%, respectively).

### PHB degradation by alkalis

We observed that a weak alkaline condition, 0.2 M NH_4_OH and NaOH at concentration lower than 0.1 M, resulted in a larger degree of PHB hydrolysis. On the other hand, cell pre-treatment by lyophilization improved the recovery yield (Table 1) and resulted in less HB and CA monomers formed when compared to fresh cells (Figure 4). This effect may be related to the crystalline state of PHB granules during treatment. In the microbial cell, PHB granules are present as hydrophobic amorphous inclusions containing 5–10% of water (Yu and Chen 2006). PHB granules at amorphous status are fragile to chemical hydrolysis. In fact, Yu and Chen (2006) and Valappil et al. (2007) suggested that PHB crystallization can increase PHB resistance to chemical treatment. PHB crystallization can be induced either by complete removal of water or by damaging the cell membrane (de Koning and Lemstra 1992), the crystallization extent being dependent on the damage level of the membrane (Kawaguchi and Doi 1990; Harrison et al. 1992). Our results confirm their observations. At weak alkaline condition and without pre-treatment, PHB in the biomass seems to maintain its amorphous status (Table 4).

PHB hydrolysis decreases the molecular weight of final products, the rate and extent of decrease being dependent on the degradation mechanism. In this study, most of the lost PHB in the supernatant could be traced back in terms of HB monomer. Furthermore, a linear relation between HB concentration and treatment time also suggested that PHB degradation occurs at the end of the polymer chain (Figure 2). This is in agreement with the observations from Yu et al. (2005) on PHB from pure cultures.

### Thermal stability

Several studies have reported molecular weight and thermal properties as an indication of PHA quality for polymer applications, for PHAs obtained from pure cultures (e.g. Kim et al. 2003, Fiorese et al. 2009, Anis et al. 2012) and from mixed cultures (summarized by Laycock et al. 2013), and for several PHA recovery and purification methods. For thermoplastic applications, thermal stability is an important parameter. An instable polymer degrades during melt processing resulting in lower molecular weight material. At a certain critical molecular weight, mechanical properties substantially deteriorate. Kanesawa and Doi (1990) studied the effect of molecular weight on mechanical properties of PHBV copolymer. They reported that the tensile strength started to deteriorate at *M*_*n*_ of 50 kg/mol and at around 20 kg/mol the sample had no strength anymore. Hablot et al. (2008) studied the effect of fermentation residues, surfactants and processing conditions on both the thermal properties and thermal degradation of PHB obtained from pure cultures by a solvent method. To our knowledge, our study provides the first data on thermal stability of PHB obtained from mixed cultures.

The sample purified by solvent showed very similar thermal stability as compared to the commercial PHB, suggesting that the quality of PHB produced by the mixed microbial culture is comparable to PHB from pure cultures. On the other hand, PHB purified by chemical treatment showed severe thermal stability deterioration. By comparing the thermal degradation rate constants of several samples relative to the commercial PHB (Table 3), this effect could be attributed to 1) residues from the chemical treatment and 2) remaining biomass impurities. The inorganics used in the treatment could either attach to the polymer chain or stay as free molecules in the polymer. In both cases, they could catalyze a polymer chain scission either via β-elimination (Kim et al. 2006) or hydrolysis mechanism (Yu and Marchessault 2000, Yu et al. 2005). These results clearly show that the choice of recovery and purification method has a large impact on material properties.

In summary, this work studied the feasibility of purifying PHB from mixed culture biomass by alkaline-based chemical treatment. The purity and recovery obtained were comparable to those reported for pure cultures. PHB losses could be attributed to hydrolysis during the chemical treatment with HB monomer as main product, also in line with what has been observed for material from pure cultures. The extent of hydrolysis can be moderated by increasing the crystallinity of the PHB granules; in this study, by either adjusting the alkali concentration, or by cell pretreatment.

The recovery and purification method had a large influence on the quality of the product for thermoplastic applications. PHB purified by solvent displayed thermal stability comparable to commercial PHB. However, PHB obtained by alkaline treatment resulted in significant thermal stability deterioration, despite of the high purity and recovery yield obtained. The quality of the product for thermoplastic applications might be improved by further optimizing the alkaline treatment process, targeting residual inorganics and biomass components. Given the potential advantages of the alkaline treatment in terms of process economics and environmental impact, it is expected that this method can be of interest for other PHB applications.
